# Standardized representation, visualization and searchable repository of antiretroviral treatment-change episodes

**DOI:** 10.1186/1742-6405-9-13

**Published:** 2012-05-03

**Authors:** Soo-Yon Rhee, Jose Luis Blanco, Tommy F Liu, Iñaki Pere, Rolf Kaiser, Maurizio Zazzi, Francesca Incardona, William Towner, Josep Maria Gatell, Andrea De Luca, W Jeffrey Fessel, Robert W Shafer

**Affiliations:** 1 Department of Medicine, Stanford University, Stanford, CA, USA; 2 Hospital Clinic Universitari-IDIBAPS, University of Barcelona, Barcelona, Spain; 3 Institute of Virology, EuResist Network GEIE, University of Cologne, Cologne, Germany; 4 Department of Medical Biotechnologies, EuResist Network GEIE, University of Siena, Siena, Italy; 5 Informasrl, EuResist Network GEIE, Rome, Italy; 6 Department of Infectious Disease, Kaiser Permanente, Los Angeles, CA, USA; 7 Institute of Clinical Infectious Diseases, Catholic University of Sacred Heart, Rome, Italy; 8 Unit of Infectious Diseases 2, University Hospital of Siena, Siena, Italy; 9 Kaiser Permanente Medical Care Program, South San Francisco, CA, USA; 10 Division of Infectious Diseases, Room S-169, Stanford University Medical Center, 300 Pasteur Drive, Stanford, CA, 94305, USA

**Keywords:** Human immunodeficiency virus, Antiretroviral treatment, Drug resistance, Clinical outcomes, XML schema, Database

## Abstract

**Background:**

To identify the determinants of successful antiretroviral (ARV) therapy, researchers study the virological responses to treatment-change episodes (TCEs) accompanied by baseline plasma HIV-1 RNA levels, CD4+ T lymphocyte counts, and genotypic resistance data. Such studies, however, often differ in their inclusion and virological response criteria making direct comparisons of study results problematic. Moreover, the absence of a standard method for representing the data comprising a TCE makes it difficult to apply uniform criteria in the analysis of published studies of TCEs.

**Results:**

To facilitate data sharing for TCE analyses, we developed an XML (Extensible Markup Language) Schema that represents the temporal relationship between plasma HIV-1 RNA levels, CD4 counts and genotypic drug resistance data surrounding an ARV treatment change. To demonstrate the adaptability of the TCE XML Schema to different clinical environments, we collaborate with four clinics to create a public repository of about 1,500 TCEs. Despite the nascent state of this TCE XML Repository, we were able to perform an analysis that generated a novel hypothesis pertaining to the optimal use of second-line therapies in resource-limited settings. We also developed an online program (TCE Finder) for searching the TCE XML Repository and another program (TCE Viewer) for generating a graphical depiction of a TCE from a TCE XML Schema document.

**Conclusions:**

The TCE Suite of applications – the XML Schema, Viewer, Finder, and Repository – addresses several major needs in the analysis of the predictors of virological response to ARV therapy. The TCE XML Schema and Viewer facilitate sharing data comprising a TCE. The TCE Repository, the only publicly available collection of TCEs, and the TCE Finder can be used for testing the predictive value of genotypic resistance interpretation systems and potentially for generating and testing novel hypotheses pertaining to the optimal use of salvage ARV therapy.

## Background

To identify determinants of successful antiretroviral (ARV) therapy in HIV-1-infected patients for whom a previous ARV treatment regimen has failed, researchers study clinical data associated with treatment-change episodes (TCEs) [1]. These studies characterize the relationship between past ARV treatments, plasma HIV-1 RNA levels, HIV-1 drug resistance genotype results, and the subsequent virological response to a salvage therapy regimen [2-8]. Such studies, however, often differ in their inclusion criteria, salvage therapy requirements, and definition of virological response.

To facilitate data sharing and analyses of combined data, we have developed a TCE XML Schema to represent treatment-change episodes. XML (Extensible Markup Language) is a markup language for encoding human and computer readable documents. An XML Schema defines constrained elements and attributes that can ensure a consistent representation of complex data. The TCE XML Schema is a richer representation of data than the flat files or spreadsheets, which form the basis for most analyses [9]. Here we collaborate with four clinics to create a public repository of 1,500 TCE XML documents represented using the TCE XML Schema (TCE Repository). To demonstrate the utility of such a repository for hypothesis generation and knowledge discovery, we analyzed a subset of the repository to obtain insights into the optimal use of second-line therapy in resource-limited settings.

We also describe two online programs that complement the TCE XML Schema: a TCE Viewer and a TCE Finder. The TCE Viewer accepts a valid TCE XML Schema document and creates a graphical depiction of the temporal relationship between ARV regimens, plasma HIV-1 RNA levels, peripheral blood CD4+ T lymphocyte counts (CD4 counts), and genotypic resistance data. The TCE Finder searches the TCE Repository according to user-defined criteria and retrieves those that meet the search criteria.

## Methods

### TCE XML schema

The TCE XML Schema elements and constraints were developed to represent the temporal relationship among ARVs, plasma HIV-1 RNA levels, CD4 counts and genotypic drug resistance data surrounding a treatment change. Each valid TCE XML Schema document must have a treatment change time point (baseline or time zero). The TCE baseline must be assigned a date or, at the very minimum, a calendar year. The preceding and subsequent data are demarcated by the number of weeks from baseline. The complete treatment history received before baseline is represented as a list of regimens, their durations, and associated plasma HIV-1 RNA levels and CD4 counts. However, if these data are not available, the XML Schema can represent the past treatment history as a list of one or more ARVs or ARV classes. Genotypic drug resistance test results are represented as nucleotide sequences or lists of amino acid mutations obtained prior to the treatment change. Optional elements include the nadir CD4 count, gender, age, ethnicity, and a metadata element for either annotating or just naming the TCE. The TCE XML Schema can be found at http://hivdb.stanford.edu/TCEs/schema/TCE.xsd.

To demonstrate the adaptability of the TCE XML Schema to different clinical environments, we collaborated with four clinics from Kaiser-Permanente Medical Care Program-Northern/Southern California, University of Barcelona and EuResist Network Database. The study was approved by the Stanford University Institutional Review Board (“Clinical Significance of HIV-1 Drug Resistance: A Clinic Based Approach”, Protocol ID: 13900).

### TCE viewer

The TCE Viewer creates a graphical depiction of a TCE (http://hivdb.stanford.edu/TCEs/cgi-bin/TCE_viewer.cgi). The TCE Viewer accepts an XML file, validates the file against the TCE Schema, and generates a graphical depiction of the TCE containing three sections: (i) a figure with the ARV regimens, plasma HIV-1 RNA levels, and CD4 counts preceding and following the treatment change; (ii) a table with one or more genotypic resistance test results preceding the treatment change; and (iii) a compressed summary of the virological and immunological responses to past ARV regimens. The TCE Viewer provides an additional mechanism of validation because many clinicians are adept at visually recognizing anomalous clinical patterns that may have resulted from data entry errors.

### TCE finder

The TCE Finder enables users to identify TCEs meeting user-defined search criteria (http://hivdb.stanford.edu/TCEs/cgi-bin/TCE_finder.cgi). The TCE Finder accepts input parameters pertaining to the ARVs used prior to the change in therapy and/or to the ARVs used for salvage therapy. A summary of the TCEs matching the input criteria are then presented to the user in a table that contains the following fields: ARVs received and genotypic resistance test results obtained prior to baseline, the salvage ARV regimen, and plasma HIV-1 RNA levels obtained while taking the salvage ARV regimen. The table also contains a thumbnail image of each TCE that links to the graphical depiction of the TCE created by the TCE Viewer.

## Results

### TCE repository

To demonstrate the ability of the TCE Schema to represent data from different clinics, we collaborated with four clinics to create a publicly available TCE repository. For the purposes of our collaboration we selected TCEs sharing each of the following criteria: (i) evidence for virological failure prior to a change in therapy defined a plasma HIV-1 RNA level of >1,000 copies/ml obtained within 8 weeks before the change; (ii) a complete list of ARVs received prior to baseline; (iii) a change in ARVs occurring within 24 weeks of a baseline genotypic resistance test; (iv) a new salvage regimen administered for at least four weeks; (v) one or more CD4 counts within 24 weeks prior to the ARV change; and (vi) two or more plasma HIV-1 RNA levels within the first 36 weeks while taking the salvage regimen.

Overall, 1,527 TCEs met the above inclusion criteria including 1,217 from Northern California, 162 from the University of Barcelona, 90 from Southern California and 58 from the EuResist Network Database. The TCEs occurred between 1998 and 2010: 555 between 1998 and 2000; 550 between 2001 and 2003; 228 between 2004 and 2006; 195 between 2007 and 2010. The median CD4 nadir was 108 (IQR: 34 to 213). The median plasma HIV-1 RNA levels and CD4 counts were 4.2 log copies/ml (IQR: 3.7 to 4.7) and 257 (IQR: 139 to 402), respectively. Patients had received a median six years (IQR: 3 to 8) of ARV therapy prior to the TCE. Previous ARVs included a median of four NRTIs, two PIs, and one NNRTI.

A complete listing of the ARVs used in the salvage regimens is shown in Table 1. Table 2 summarizes the ARV class combinations comprising the salvage therapy regimens: (i) 1,382 regimens (denoted in Table 2 as Type 1 regimens) comprised combinations of the first three approved ARV classes: NRTIs, NNRTIs, and PIs; (ii) 145 regimens (denoted in Table 2 as Type 2 regimens) contained at least one of the newer classes including the integrase inhibitor, raltegravir (RAL), fusion inhibitor, enfurvirtide (ENF), and CCR5 antagonist, maraviroc (MVC).

**Table 1:** Antiretrovirals (ARVs) Used For Salvage Therapy in the 1,527 Treatment Chance Episodes (TCEs)^*^

**ARV Class**	**PIs**^**†§**^	**NNRTIs**	**NRTIs**	**Other**
ARVs	LPV/r (401)	EFV (399)	3TC (654)	RAL (104)
	FPV/r (187)	NVP (164)	TDF (604)	MVC (22)
	SQVr (158)	ETR (28)	d4T (587)	ENF (48)
	IDV/r (129)	DLV (15)	ddI (511)	
	ATV/r (120)		ABC (435)	
	NFV (100)		AZT (260)	
	DRV/r (81)		FTC (185)	
	TPV/r (27)			
Total	1,203	606	3,236	174

^*^The number within parenthesis following an individual ARV indicates the number of TCEs in which the ARV was included in the salvage therapy regimen.

^**†**^ “/r” indicates ritonavir-boosting.

^§^Included among the 187 TCEs listed as having received FPV/r are 76 who received unboosted FPV or APV; Included among the 158 TCEs listed as having received SQV/r are 23 who received unboosted SQV; Included among the 129 TCEs listed as having received IDV/r are 34 who received unboosted IDV; Included among the 120 TCEs listed as having received ATV/r are 4 who received unboosted ATV.

Abbreviations: *PI* protease inhibitor; *NRTI* nucleoside RT inhibitor; *NNRTI* non-nucleoside RT inhibitor; *LPV* lopinavir; *FPV* fosamprenavir; *SQV* saquinavir; *IDV* indinavir; *ATV* atazanavir; *NFV* nelfinavir; *DRV* darunavir; *TPV* tipranavir; *EFV* efavirenz; *NVP* nevirapine; *ETR* etravirine; *DLV* delavirdine; *3TC* lamivudine; *TDF* tenofovir; *d4T* stavudine; *ddI* didanosine; *ABC* abacavir; *AZT* zidovudine; *FTC* emtricitabine; *RAL* raltegravir; *MVC* maraviroc; *ENF* enfuvirtide.

**Table 2:** Summary of the ARV Class Combinations Comprising the Salvage ARV Regimens

**Type 1 Regimens NRTIs, NNRTIs, and/or PIs**		**Type 2 Regimens Raltegravir (RAL), enfuvirtide (ENF), and/or Maraviroc (MVC)**	
ARV Class Combination	No. TCE	ARV Class Combination	No. TCE
2 NRTIs + PI/r	364	≥1NRTI + PI/r + RAL	37
≥3 NRTIs + PI/r	194	≥1NRTI + PI/r + ENF	30
2 NRTIs + NNRTI	149	≥1NRTI + NNRTI + PI/r + RAL	13
1 NRTI + NNRTI + PI/r	102	≥1NRTI + NNRTI + PI/r + ENF	7
2 NRTIs + NNRTI + PI/r	90	≥1NRTI + RAL	7
2 NRTIs + PI	90	≥1NRTI + PI/r + RAL + ENF	6
≥3 NRTIs + NNRTI	79	PI/r + RAL	5
≥3 NRTIs	62	PI/r + RAL + MVC	5
1 NRTI + NNRTI + PI	49	≥1NRTI + NNRTI + RAL	4
1 NRTI + PI/r	45	≥1NRTI + PI/r + RAL + MVC	4
2 NRTIs + NNRTI + PI	44	≥1NRTI + RAL + MVC	4
≥3 NRTIs + PI	36	NNRTI + PI/r + RAL	3
≥3 NRTIs + NNRTI + PI/r	25	≥1NRTI + NNRTI + PI + ENF	2
NNRTI + PI	19	≥1NRTI + NNRTI + RAL + ENF	2
≥3 NRTIs + NNRTI + PI	9	≥1NRTI + PI/r + MVC	2
PI/r	3	≥1NRTI + PI/r + MVC + ENF	2
		NNRTI + RAL + MVC	2
Miscellaneous	22	Miscellaneous	10
Total	1,382	Total	145

Fewer than 10 individuals received a double-boosted PI either as part of a Type 1 or Type 2 Regimen. The PI component in these individuals was denoted here as PI/r.

To avoid subdividing the Type 2 Regimens into too many small categories, no distinction was made based on the number of NRTIs in the regimen.

Abbreviations: *NRTIs* nucleoside RT inhibitors, *NNRTIs* non-nucleoside RT inhibitors, *PIs* protease inhibitors, *PI/r* ritonavir-boosted PIs.

The median duration of the salvage therapy regimen was 52 weeks (IQR: 38 to 52). Plasma HIV-1 RNA levels following the ARV change were available a median of every 13 weeks. One or more plasma HIV-1 RNA levels were available in 91% of TCEs during the 8 to 16 week window following the change in therapy, in 83% of TCEs during the 16 to 36 week window, and in 58% of TCEs during the 36 to 52 week window. Two or more plasma HIV-1 RNA levels were available in 49% of TCEs following the change in therapy during the 8 to 16 week window, in 37% of TCEs during the 16 to 36 week window, and in 17% of TCEs during the 36 to 48 week windows.

Of the TCEs for which two or more plasma HIV-1 RNA levels were available during the 16 to 36 week window (n = 562), there was a significant increase over time in the proportion of TCEs for which two or more consecutive plasma HIV-1 RNA levels were below the level of quantification: 24% of the 140 TCEs occurring between 1998 and 1999, 38% of 186 TCEs occurring between 2000 and 2001, 50% of 139 TCEs occurring between 2002 and 2004, 69% of 97 TCEs occurring between 2005 and 2010 (OR: 1.3; *p* < 0.0001).

**Figure 1:**
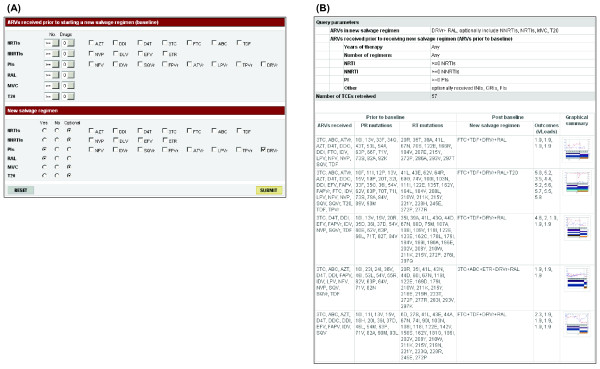
**Treatment-Change Episode (TCE) Finder.** TCE Finder user interface including the input form (A) and output showing the summary of the TCEs matching the input criteria (B). The example shown here is searching TCEs containing DRV/r and RAL in the salvage regimen. Fifty-seven TCEs that met the input criteria were retrieved and the first five are shown in (A). For each TCE retrieved, the output table contains the following fields: ARVs received prior to baseline, genotypic resistance test results at baseline, plasma HIV-1 RNA levels obtained following the change in therapy, and a thumbnail image of each TCE that links to the graphical depiction of the TCE created by the TCE viewer. The graphical depiction of the last TCE in the output is shown in Figure 2.

**Figure 2:**
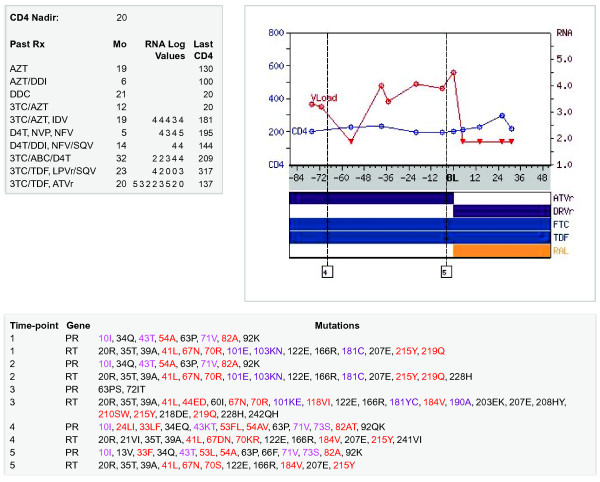
**Treatment-Change Episode (TCE) Viewer.** TCE Viewer plots (i) antiretroviral (ARV) regimens, plasma HIV-1 RNA levels, and CD4 counts surrounding a treatment change (upper right); (ii) genotypic resistance test results (bottom); and (iii) a summary of the past ARV history (upper left). ARVs are shown beneath the TCE timeline; plasma HIV-1 RNA levels in log copies/ml are shown in red; and CD4 counts are shown in blue. Plasma HIV-1 RNA levels below the limits of quantification are indicated by inverted triangles. Genotype times are indicated by numbered vertical dotted lines. Genotypic resistance test results list amino acid differences from the consensus B protease and RT sequences. Nucleoside-, nonnucleoside, and protease inhibitor resistance mutations are colored. The past history summary shows the CD4 nadir and a list of past ARV regimens. For each past regimen, the plasma HIV-1 RNA levels rounded to the nearest log_10_ value and the last CD4 count are shown.

The TCE XML documents have been placed in a publicly available repository found on the following web page: http://hivdb.stanford.edu/TCEs/. The TCE Repository contains three primary functions. First, users can employ the TCE Finder to identify TCEs matching specific criteria (Figure 1) and examine the virological responses associated with the TCEs. Second, users can obtain a graphical depiction of their own TCEs by submitting a TCE XML document to the TCE Viewer (Figure 2). Third, users can download the entire set of TCE XML documents in a compressed file format or browse each TCE document using the TCE Viewer.

### Virological response to initial PI and NNRTI therapy: insights from the TCE repository

**Figure 3:**
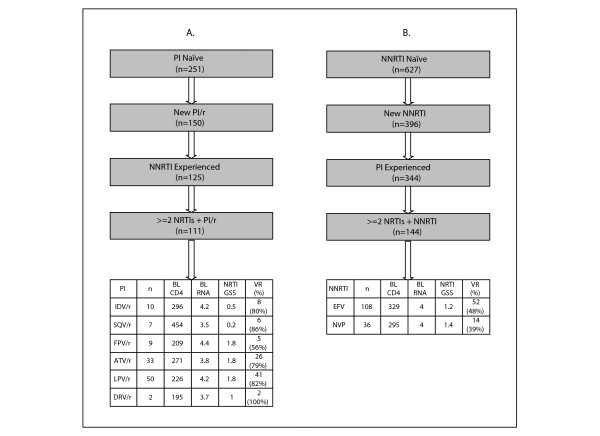
**Treatment change episodes (TCEs) of (A) PI-naïve and NRTI/NNRTI-experienced patients receiving a new regimen containing a ritonavir-boosted PI and (B) NNRTI-naïve and NRTI/PI-experienced patients receiving a new regimen containing an NNRTI.** The flow diagrams illustrate the process by which the patients meeting the selection criteria where extracted from the TCE Repository. To reflect the clinical reality in resource limited settings, patients receiving an integrase inhibitor, fusion inhibitor, or maraviroc were excluded. The tables beneath the flow diagrams contain the numbers of patients (n) according to the specific PI/r (A) or NNRTI (B) and the proportions with virological suppression to <50 copies/ml within 48 weeks (virological response; VR). The NRTI GSS was derived using the Stanford HIVdb algorithm (http://hivdb.stanford.edu; accessed January 25, 2012). Abbreviations: PI - protease inhibitor; PI/r - ritonavir-boosted PI; NRTI - nucleoside RT inhibitor; NNRTI - non-nucleoside RT inhibitor; LPV - lopinavir; FPV - fosamprenavir; SQV - saquinavir; IDV - indinavir; ATV - atazanavir; DRV - darunavir; EFV - efavirenz; NVP – nevirapine.

One of the most pressing clinical challenges in resource-limited settings is the design of salvage therapy strategies for patients developing virological failure following an initial NRTI/NNRTI-containing regimen or, less commonly, an initial NRTI/PI-containing regimen. Figure 3A illustrates that the TCE XML Repository contains 111 NRTI/NNRTI-experienced but PI-naïve patients who received salvage therapy with a ritonavir-boosted PI and an optimized NRTI backbone. Figure 3B illustrates that the Repository contains 144 NRTI/PI-experienced but NNRTI-naïve patients who received salvage therapy with an NNRTI and an optimized NRTI backbone.

The proportion of patients attaining virological suppression (<50 copies/ml) in the first 6 to 12 months of therapy was significantly higher in those receiving salvage therapy with a boosted PI (88/111, 79%) compared with an NNRTI (66/144, 46%; *p* < 0.001). The drug class used for salvage therapy (boosted PI vs. NNRTI) remained significant in a multivariate analysis that controlled for baseline CD4 count, plasma HIV-1 RNA level, calendar year, and the expected activity of the optimized NRTI backbone (i.e., the NRTI genotypic susceptibility score, GSS). Among those receiving boosted PIs, the proportions of responders were similar in those receiving atazanavir (26/33, 79%) compared with lopinavir (41/50, 82%). However, the mean baseline CD4 count was higher (343 vs. 263) and the mean baseline plasma HIV-1 RNA level was lower (3.9 vs. 4.2 log copies/ml) in those receiving atazanavir. Among those receiving NNRTIs, the proportions of responders were similar in those receiving efavirenz (52/108, 48%) compared with nevirapine (14/36, 39%). The mean baseline CD4 count and plasma HIV-1 RNA level were also similar in those receiving efavirenz compared with nevirapine (323 vs. 310; 4.1 vs. 4.2 log copies/ml).

However, in the pooled analyses of the 144 NNRTI-naïve patients receiving an NNRTI, the number of NRTIs included in the optimized backbone was significantly associated with virological suppression: 27 of 44 patients (61%) who received three or four NRTIs attained virological suppression compared with 39 of 100 patients (39%) who received two NRTIs (*p* = 0.02; chi-square test). Neither the number of NRTIs nor the NRTI GSS was significantly associated with response to the PI-naïve patients receiving a boosted PI and an optimized NRTI backbone.

## Discussion

The Department of Human Health Services (DHHS) and the WHO [10] have guidelines on which ARV regimens to use for initial and second-line therapy of HIV-1-infected patients. However, many clinical scenarios are not addressed by these guidelines including the management of (i) patients who began ARV therapy with suboptimal regimens – a problem particularly common in the U.S., Europe in the past, and many middle income countries where previously available ARVs were considerably less potent and drugs were used as they became available rather than as part of a national treatment program, (ii) patients with transmitted resistance, and (iii) heavily treated patients and patients whose viruses have complex patterns of drug-resistance mutations.

The difficulty of recommending therapy for such patients has motivated researchers to study how pre-treatment characteristics influence the response to a change in ARV therapy. Indeed, there have been many studies correlating the presence of baseline ARV-resistance mutations with the response to a new ARV regimen while accounting for essential covariates such as past treatment history, baseline plasma HIV-1 RNA levels, and baseline CD4+ counts. Such studies, however, often differ in their inclusion criteria (i.e. past ARV treatments, timing of plasma HIV-1 RNA levels and genotypic resistance data), salvage therapy requirements [11-18], and definition of virological response. For example, some studies define virological response by the extent of reduction in plasma HIV-1 RNA levels, whereas others define it as the suppression of plasma HIV-1 RNA levels below the limits of quantification [19,20]. Most of studies have examined plasma HIV-1 RNA levels at fixed time points, whereas an approach based on time to virological failure has been recently proposed [21].

A standardized representation of data such as that found in the TCE XML Schema makes it possible to apply uniform inclusion and virological endpoint criteria across TCEs from different studies. The combined data can be analyzed for three purposes (i) to reproduce prior results, (ii) to apply and test new analytic methods, and (iii) to generate or test new hypotheses. Many tools are available to validate and transform the contents of XML Schema documents. XML Schemas therefore ensure that the data are represented consistently and can be readily integrated into different applications.

Studies of TCEs typically do not analyze the complete treatment history of a patient. Rather these studies parameterize essential features of the patient’s past ARV exposures. This condensed treatment history combined with the response to a new therapy was called a “treatment-change episode” (TCE) by Larder et al. of the Resistance Database Initiative (RDI) [22]. The TCE XML Schema is therefore much less complex than the relational database implemented by the HIV Cohort Data Exchange Protocol (HICDEP) [23]. Moreover, the fact that the TCE XML Schema does not require demographic or epidemiologic data and allows relative (rather than absolute) dates makes it impossible to identify individual patients or clinics [24].

The TCE XML suite comprises four medical informatics tools: (1) The XML Schema; (2) The TCE Viewer, an online program that creates a graphical representation of data in the XML document; (3) The TCE Repository, which provides the proof-of-concept that the TCE XML Schema can be used to exchange data from multiple clinics; and (4) The TCE Finder, a search engine to identify TCEs meeting specific criteria. The TCE XML suite is useful for comparing genotypic resistance interpretations and hypothesis generation and testing. It should therefore be distinguished from ongoing projects designed to optimize therapy for individual patients such as RDI’s HIV Treatment Response Prediction System (TREPS) [22] and Genafor’s Theo [25]. However, because the data in the TCE Repository is publicly available it can be used to increase the training sets for machine learning systems such as Theo and TREPS.

Despite its nascent stage, the TCE Repository has already been shown to be useful for comparing different genotypic resistance test interpretation systems. Specifically, 734 of the TCEs were previously used in a study comparing the predictive value of three algorithms [26]. Without such a repository, comparisons of genotypic resistance interpretation systems can be performed solely by using proprietary datasets. In addition, we demonstrate here that the TCE Repository makes it possible to generate novel hypotheses that that may be relevant to salvage therapy in resource-limited regions. Indeed, at least one other research team has proposed the use of three rather than two NRTIs for certain salvage therapy scenarios in regions without access to newer ARV classes [27]. However, considering the large number of covariates associate with treatment response, very large numbers of TCEs will be required to adequately test novel hypotheses.

Although the XML Schema and Viewer are useful to individual research groups and collaborations, the usefulness of the Finder and Repository depends on the willingness of researchers to contribute data to this effort. Therefore, we have collaborated with four research groups to demonstrate the utility of the XML suite of applications for collaboration between multiple clinics. We are continuing to work with clinics in North America, Spain, and the EuResist Network Database to expand the Repository with TCEs that are relevant to resource limited regions (i.e. the regimens are confined primarily to NRTI, NNRTIs, and PIs) and with TCEs involving the use of more recently approved ARV classes including the integrase inhibitors and maraviroc.

## Conclusions

The TCE Suite of applications – the XML Schema, Viewer, Finder, and Repository – addresses several major needs in the analysis of predictors of virological response to ARV therapy. The TCE XML Schema facilitates data sharing for generating and testing new hypotheses. The TCE Viewer helps users validate the temporal relationship between different data elements and it can be a useful teaching tool. The TCE Finder is an application designed for researchers who do not want to download the entire TCE repository but who would rather examine the solely the clinical data of patients sharing similar ARV treatment and genotypic resistance characteristics. The TCE Repository is the largest collection of publicly available TCEs. It is already useful for comparing the predictive value of genotypic resistance interpretation systems. As it increases in size it will become an increasingly useful resource for hypothesis generation and knowledge discovery.

## Authors’ contributions

SYR and RWS designed the study and wrote the manuscript. JLB, RK, MZ, WT, JG, ADL and WJF collected and annotated the clinical data. SYR, TFL, IP and FI created and implemented procedures for transferring data from clinical databases to the TCE XML Schema documents. MZ, ADL and WJF also contributed to drafting the manuscript. All authors read and approved the final manuscript.

## Competing interests

The authors do not have a commercial or other association that might pose a competing interest.
